# Phlegmonous Gastritis as the First Presentation of Acute Leukemia: A Case Report

**DOI:** 10.7759/cureus.83156

**Published:** 2025-04-28

**Authors:** Mohammed A Alkubaisi, Nader K Mohammed, Ibrahim S Ibrahim, Hamza I Rashid

**Affiliations:** 1 Medicine, Peterborough City Hospital, Peterborough, GBR; 2 Gastroenterology, Peterborough City Hospital, Peterborough, GBR; 3 Acute Internal Medicine, Peterborough City Hospital, Peterborough, GBR

**Keywords:** gi endoscopy, infectious disease, leukemia, phlegmonous gastritis, radiology

## Abstract

Phlegmonous gastritis (PG) is a serious and severe suppurative gastric infection. Diagnosis is delayed due to its uncommon and non-specific clinical presentation. This led to a high mortality rate; however, early treatment with broad-spectrum antibiotics can reduce mortality and complications. We describe a case of a 56-year-old patient who presented with severe abdominal pain, vomiting, and pyrexia. Blood tests showed pancytopenia, and she was newly diagnosed with acute myeloid leukemia (AML) on this admission. Her computed tomography (CT) and endoscopic evaluation showed thickening and swelling of the gastric wall consistent with PG. She responded well to conservative treatment, and she is on regular follow-up as an outpatient. This case highlights the importance of suspecting PG in severe abdominal pain and neutropenia. This will reduce the complications, mortality, and requirement for surgical intervention.

## Introduction

Phlegmonous gastritis (PG) is a rare, severe infection of the gastric wall, with a high mortality rate of up to 40%. Pathophysiology is not fully understood, but it is thought to involve bacterial invasion of the gastric wall mucosa and reaching the muscular layer either directly or from bacteremia. These organisms may be gas-forming, and this leads to mucosal swelling, inflammation, and intramural gas [1,2]. Clinical presentation is non-specific and may include severe epigastric abdominal pain, vomiting, and fever; however, purulent vomiting is characteristic [3]. It is more common in patients with predisposing factors such as immunodeficiencies and alcoholism; however, it can happen in patients with no risk factors [4]. A computed tomography (CT) scan can show diffuse thickening and edema of the gastric wall [1,5]. Gastric endoscopy can show an inflamed gastric wall and occasionally purulent discharge and hemorrhage. Gastric biopsy is crucial for obtaining culture and ruling out gastric pathology [4]. PG requires early, broad-spectrum antibiotics to reduce the mortality. Surgical intervention may be needed for possible complications like perforation and peritonitis [5]. We report a patient who developed PG in the setting of the first presentation of acute leukemia, was successfully treated with systemic antimicrobial therapy and chemotherapy, and does not require total parenteral nutrition or surgical intervention.

## Case presentation

A 56-year-old female presented to the emergency department with very severe abdominal pain for three days before admission. The pain was in the upper middle abdomen, and the score was nine out of 10. Pain was described as a dull ache that was radiating to the right upper quadrant. She also described repeated green-colored vomitus and a fever. She has a background of type 2 diabetes mellitus (T2DM) and hypertension (HTN). She is a non-smoker and does not drink alcohol. Her medications were insulin (Lantus), metformin 1 g twice daily, amlodipine 5 mg once daily, and atorvastatin 20 mg once daily. On admission, her temperature was 39 °C, blood pressure was 120/80 mmHg, heart rate was 90 beats per/minute, and oxygen saturation was 97% on air. She was also observed to be dehydrated, and on abdominal examination, she had right upper quadrant and epigastric tenderness. She was also Murphy’s sign positive. Bloods were taken and initial treatment was prescribed, including intravenous (IV) fluids, analgesia, and co-amoxiclav. The blood results are shown in Table 1.

**Table 1:** Blood results on admission, after six days and after 30 days. CRP: C-reactive protein, WBC: white blood cell

	Result on admission	After six days	After 30 days	Normal ranges
CRP	103	199	53	<5 mg/L
Amylase	45			<100 U/L
Coagulation profile	Unremarkable			
WBC	3.1	9.2	14.3	4-11 10^9^/L
Hemoglobin	94	59	84	115-165 g/L
Platelets	72	80	211	150-400 10^9^/L
Neutrophils	< 0.1	< 0.1	8	10^9^/L
Lymphocytes	0.5	0.4	1.4	1.4-4.8 10^9^/L
Creatinine	46	51	98	45-84 umol/L
Glucose	14.6			3.9-5.4 mmol/L
Blood film	Numerous blast cells			
Blood culture on admission	Negative			
Blood ketones	0.4 mmol/L			

**Figure 1:**
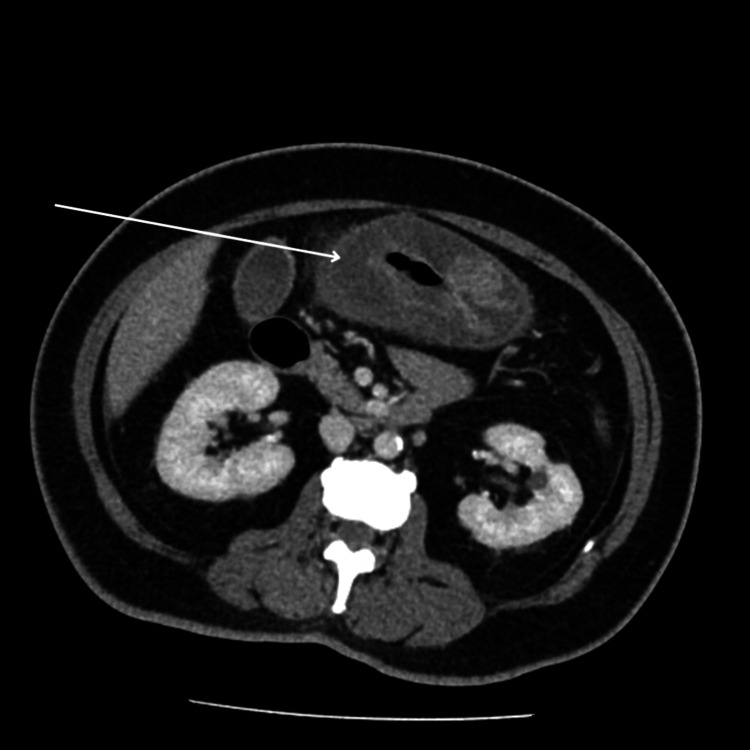
CT images detailing diffuse thickening of the gastric wall (white arrow).

Given her high temperature, severe abdominal pain, and low WBC, a CT scan with contrast of the abdomen and pelvis was requested. The CT showed diffuse thickening of the stomach wall (Figure 1).

**Figure 2:**
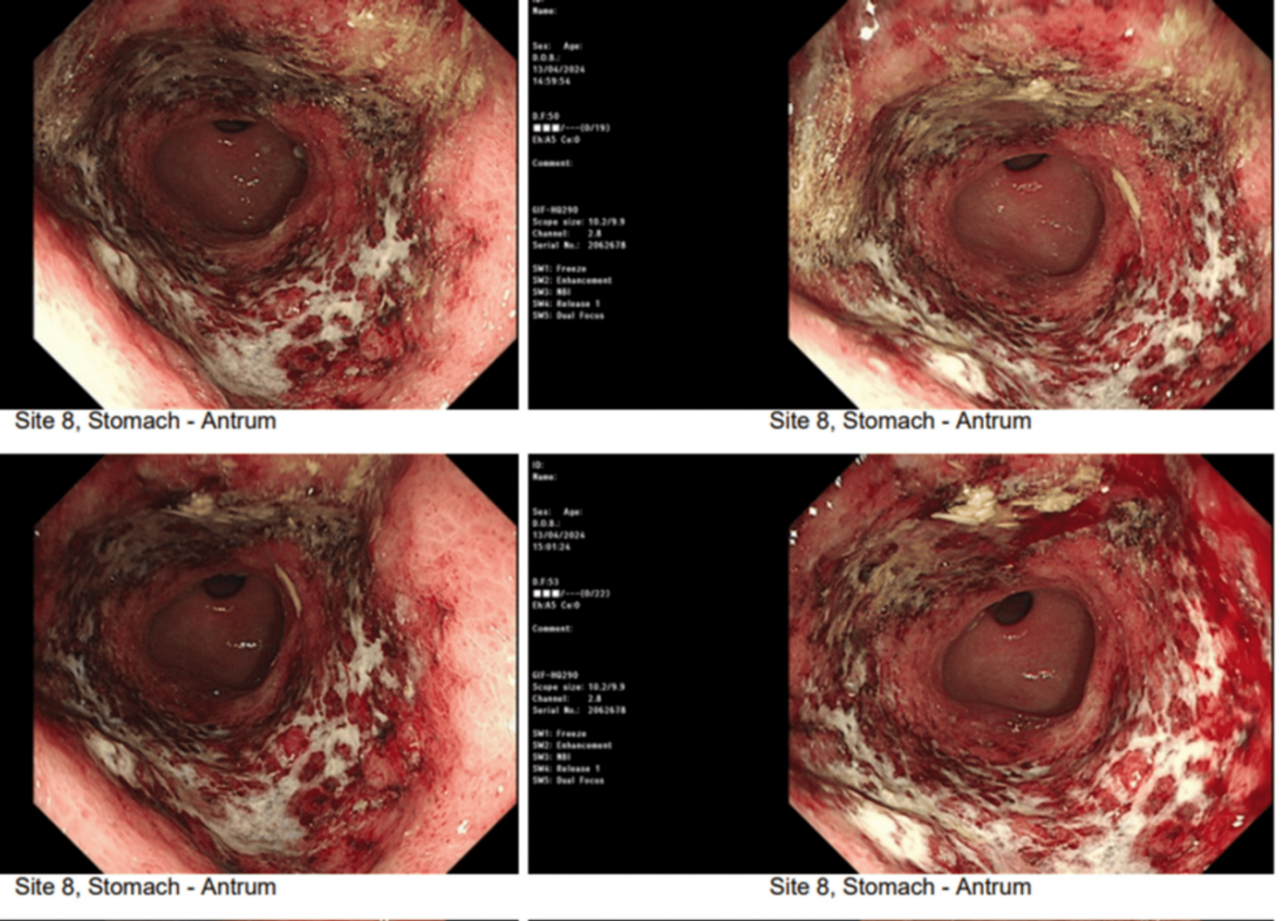
Images obtained during oesophago-gastro-duodenoscopy (OGD) demonstrating white patches on the esophagus (Candida) and necrotic and ulcerative tissue in the stomach.

Oesophago-gastro-duodenoscopy (OGD) showed multiple adherent white patches in the esophagus, which were consistent with esophageal candidiasis. Gastric ulceration and an inflamed gastric wall, which bled easily upon contact, were also observed (Figure 2). Flow cytometry of peripheral blood confirmed acute myeloid leukemia (AML) with 87% blast cells.

**Figure 3:**
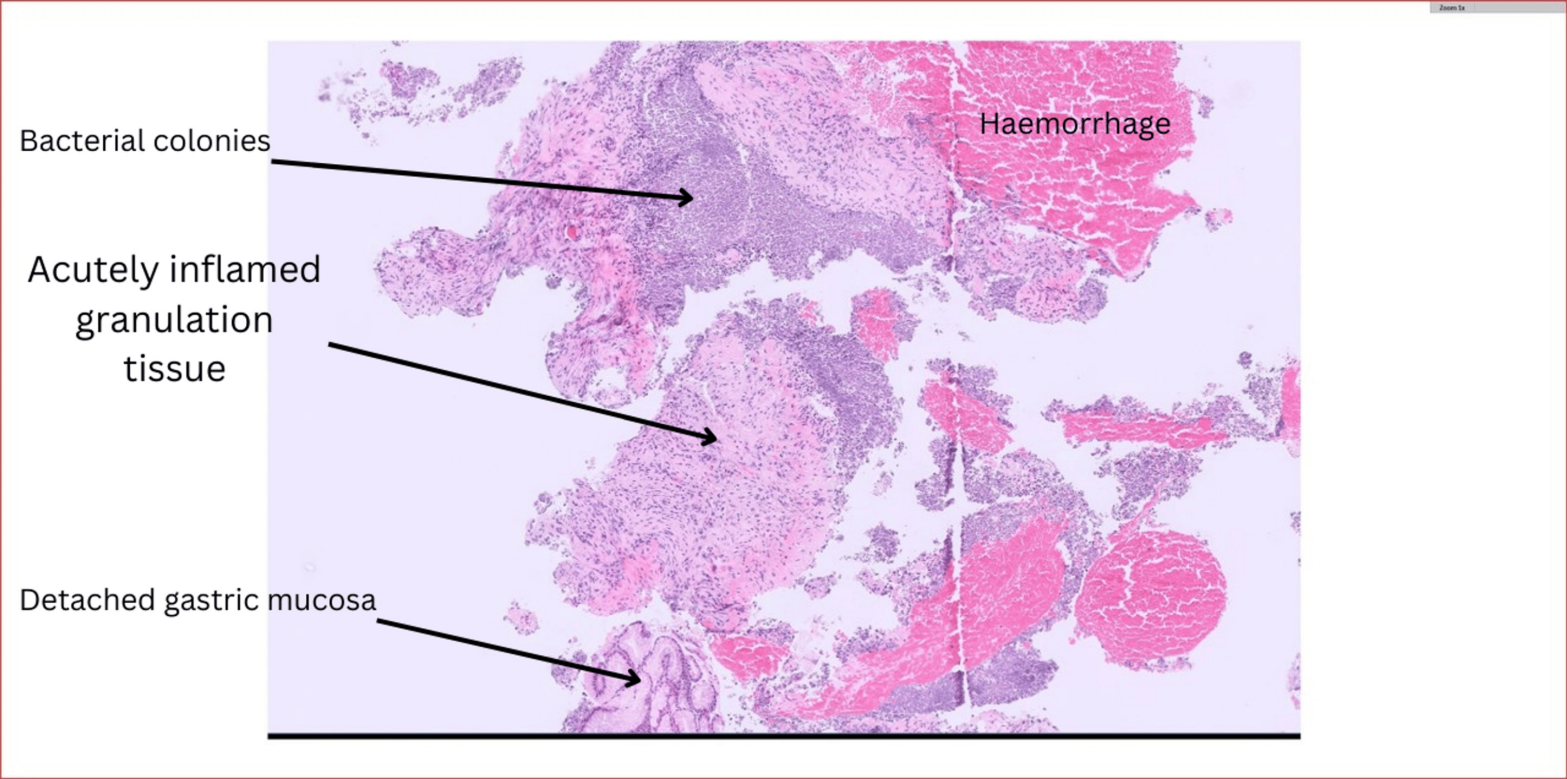
Gastric antrum detailing the granulation tissue ulcer bed coated in bacteria.

A biopsy was obtained from the stomach. The biopsy reported acutely inflamed granulation tissue and some bacterial overgrowth. There was no dysplasia or evidence of malignancy, and the appearances are compatible with phlegmonous gastritis (Figure 3). Another gastric biopsy showed abundant fungal hyphae.

Case progression

**Figure 4:**
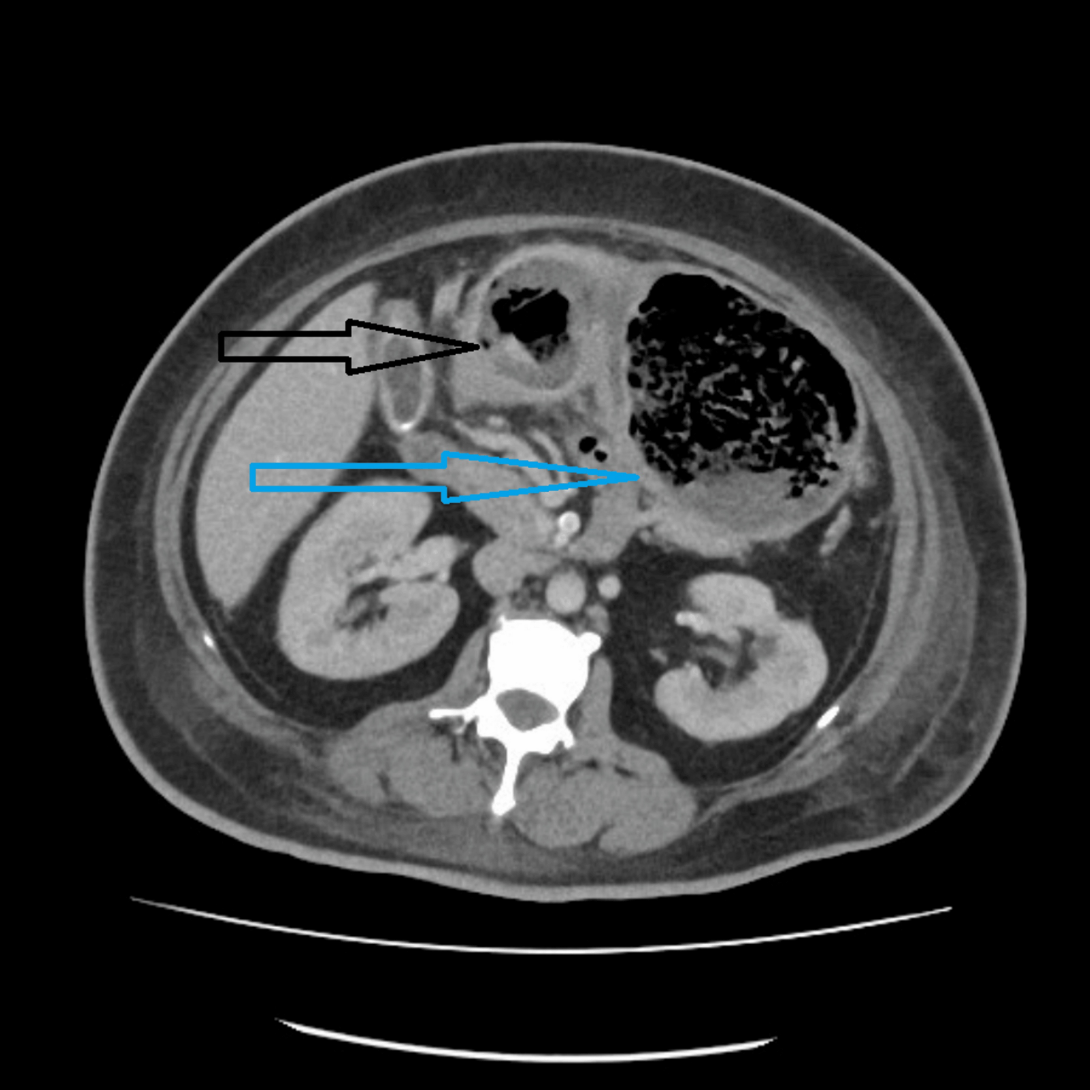
CT images detailing resolution of gastric body wall thickening (blue arrow) and pyloric intramural air hypodensities (black arrow).

She stayed in the hospital for 34 days and required admission to the intensive care unit due to oxygen requirements. A repeated CT abdomen after 14 days showed reduced gastric body wall thickness but showed gases in the pyloric wall (Figure 4).

**Figure 5:**
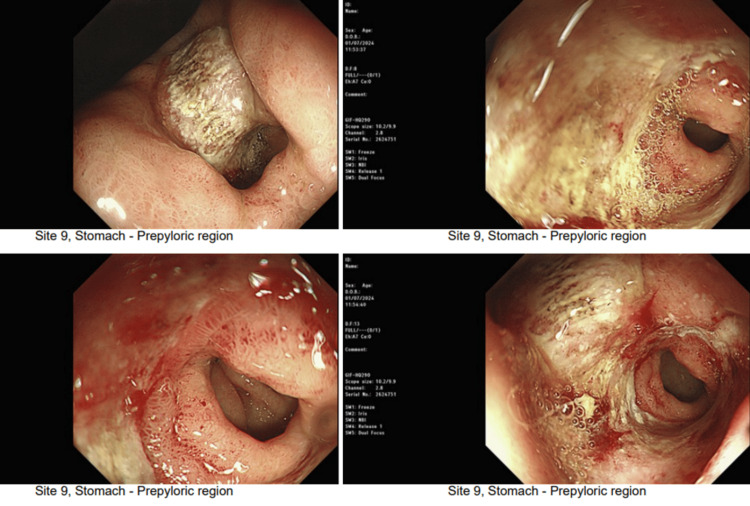
Images obtained on repeat oesophago-gastro-duodenoscopy (OGD) showing an improved gastric wall.

Her second OGD was performed three months after her first presentation. The OGD showed a normal esophagus and improved gastric appearance; however, there was a suspicious hard pre-pyloric lesion, and multiple biopsies were obtained (Figure 5). 

## Discussion

PG is quite uncommon, with one case annually reported. Due to the development of antimicrobials, the mortality rate decreased significantly from 92% to 40%. It is more common in males than females, with a ratio of 2:1, and more commonly affects the ages 50-70 [6]. The pathogenesis of PG is not fully understood. Still, several routes are suggested, such as a direct invasion from the gastric wall; blood spread from another organ; ingestion of the pathogen, such as in the respiratory tract infection; or lymphatic spread from cholecystitis and peritonitis [7]. There are two main types of PG: localized PG affects mainly the antrum, and diffuse PG affects the whole stomach. PG can also affect both the stomach and esophagus at the same time [5,8]. The presentation of PG includes non-specific symptoms such as abdominal pain, high temperature, nausea, and vomiting. The presence of vomiting containing pus can be characteristic [3]. The risk factors for PG are old age, DM, immunocompromise, peptic ulcers, septicemia, and instrumental use such as OGD and nasogastric tube. However, it can happen in patients with no risk factors [3,5]. *Streptococcus* is the cause in two-thirds of the cases, and one-third is polymicrobial. Other organisms are *Staphylococcus*, *Escherichia coli*, *Proteus*, and *Haemophilus influenzae *[6]. Early radiological studies are very crucial for the diagnosis. Early CT finding is diffuse thickening of the gastric wall. Later CT scans may show low-intensity areas within the gastric wall (indicative of an abscess) and also may show intraluminal gases within a thick wall, and this is called emphysematous gastritis [2,5]. The typical features of OGD in PG are red, swollen stomach folds and mucosa, superficial ulcerations, and attached purulent mucus. However, these features are non-specific to PG [4]. Possible complications are perforation, peritonitis, gastric stricture, sepsis, and death. These complications are extremely high in patients with hematologic malignancies due to low WBC [1].

The differential in our case was acute cholecystitis, pancreatitis, gastritis, peptic ulcer, diabetic ketoacidosis (DKA), and lower respiratory tract infection (LRTI). Her blood tests showed a normal amylase level, and for that, pancreatitis was excluded. Given a normal oxygen level with a normal respiratory rate, LRTI was excluded. DKA was excluded due to normal ketone levels. Blood tests showed pancytopenia with low hemoglobin, WBC, and platelets. A hematology opinion was obtained, which later confirmed the diagnosis of AML by blood flow cytometry, and the patient was started on chemotherapy. The CT scan, OGD, and gastric biopsy showed features of PG. Unfortunately, no culture was obtained from the gastric antrum.

Due to the high mortality and complications, broad-spectrum antimicrobials should be started. Surgery may be needed in the case of a contracted stomach, peritonitis, or perforation [1]. To date, there are no specific measures for continuing the antibiotics after clinical improvement. Hence, follow-up should be completed with OGD to rule out any remaining ulcer or tumor [3].

Our patient was started on IV piperacillin with tazobactam when blood showed neutropenic sepsis. The antifungal AmBisome was added, and she was started on chemotherapy for her AML. Four days later, due to no improvement in the clinical features and inflammatory markers, antibiotics were switched to meropenem, and the AmBisome was switched to caspofungin for two weeks due to acute kidney injury. The blood culture after 14 days of IV antibiotics was positive for *E. coli *(*E. coli *AMPC-producing organism). On the advice of Microbiology, antibiotics were switched to ciprofloxacin, metronidazole, and teicoplanin. During her stay, she required multiple blood transfusions to maintain her hemoglobin above 80 g/L and platelets above 20 x 10^9^/L. After these changes in her treatment, she clinically improved. She had a further follow-up as an outpatient OGD, which showed improvement from the previous gastric findings.

## Conclusions

Doctors should be highly suspicious of PG in leukemia patients who present with acute unwellness and abdominal pain. An abdominal CT scan is necessary to help with diagnosis. OGD is also important for obtaining a biopsy and culture to guide the antimicrobial treatment. In such cases, early antibiotics are mandatory to reduce the complications and mortality. Moreover, early discussion with the microbiology team for appropriate advice on antimicrobials is vital. Further follow-up is needed to rule out underlying malignancy or ulcer.
